# Nonsmall Cell Lung Cancer Therapy: Insight into Multitargeted Small-Molecule Growth Factor Receptor Inhibitors

**DOI:** 10.1155/2013/964743

**Published:** 2013-07-01

**Authors:** Mridul Roy, Yu-Hao Luo, Mao Ye, Jing Liu

**Affiliations:** ^1^Molecular Biology Research Center, School of Life Science and State Key Laboratory of Medical Genetics of China, Central South University, Changsha, Hunan 410078, China; ^2^Molecular Science and Biomedicine Laboratory, State Key Laboratory for Chemo/Biosensing and Chemometrics, College of Biology, College of Chemistry and Chemical Engineering, Hunan University, Changsha 410082, China

## Abstract

To date, lung cancer is the leading cause of cancer-related death worldwide, among which nonsmall cell lung cancer (NSCLC) comprises about 85%. Taking into account the side effects of surgery, radiation, platinum-based doublet chemotherapy, and the growth self-sufficiency characteristic of cancer cells, drugs have been discovered toward growth factor receptor (GFR) to treat NSCLC. As expected, these drugs provide a greater benefit. To increase the efficacy of such growth factor receptor tyrosine kinase inhibitors (RTKIs), coinhibition of GFR signaling pathways and combination of inhibitors along with radiation or chemotherapy have drew intense insight. Although clinical trials about single-agent RTKIs or their combination strategies suggest their increase potency against cancer, they are not beyond adverse effects, and sometimes the effects are more deadly than chemotherapy. Nevertheless the hope for RTKIs may be proved true by further researches and digging deep into cancer therapeutics.

## 1. Introduction

Worldwide, the impact of lung cancer is enormous making it the leading cause of cancer-related mortality [1]. Nonsmall cell lung cancer (NSCLC) accounts for approximately 85% of all cases of lung cancer [2]. Standard first-line treatment options for NSCLC depend on disease and patient characteristics and may include surgery, platinum-based doublet chemotherapy, and targeted therapies [3]. Although surgical resection is curative if diagnosis occurs at early stage I or stage II disease, almost half of all newly diagnosed patients are with advanced-stage disease and candidates for palliative systemic therapies. Chemotherapy for nonsmall cell lung cancer (NSCLC) has shown modest improvements in patients with stages II and IIIA NSCLC with an improvement in both overall survival (OS) and quality of life [4, 5]. This first-line, platinum-based doublet chemotherapy regimen is associated with modest clinical benefits, but it has significant toxicities [6]. Furthermore chemotherapy combinations for more advanced disease have shown to convey no benefit on overall survival or quality of life beyond 4–6 cycles [7, 8]. As knowledge about molecular abnormalities that drive cell growth and proliferation for lung cancers has grown and as NSCLC currently has a 5-year survival rate of less than 20% [9], there is clearly a need for the development of more effective therapies. According to Hanahan and Weinberg [10], the cell surface receptors that transduce signals into the cell are the targets of deregulation during tumor progression resulting in self-sufficiency in growth signal, one of the major hallmarks for cancer cells. Growth factor receptors (GFRs) are overexpressed in many cancers which may enable the cancer cell to become hyperresponsive to ambient level of growth factors and even ligand-independent signaling. This observation provides the rationale for the interest of research to develop anti-GFR compounds.

Although treatments, which target individual pathway, have showed clinical successes, the ability of tumor cell to develop resistance to circumvent inhibition of a single signaling pathway drives the urge to target multiple signaling pathways. Therapeutic approaches to inhibit multiple pathways using multiple single-targeted agents may help to maximize the suppression of oncogenic processes involved in disease progression. Using a single multitargeted agent, rather than to use multiple single agents, to individually attack multiple targets is an alternative strategy. In this study we reviewed the mechanisms that lung cancer cells carry out for growth, proliferation, angiogenesis, and metastasis by using GFRs, and how they are being cotargeted by small-molecule inhibitors and current trial use of these molecules as treatment of NSCLC at I/II/III phases. In addition we reviewed the studies about the combination of targeting agents with other molecules to achieve a better efficacy.

## 2. Role of GFRs on NSCLC

**Figure 1:**
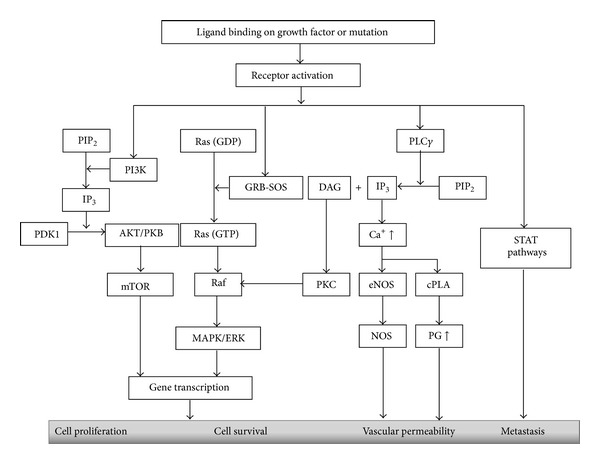
Possible pathways for growth factor mediated tumorigenesis. Activation of receptor can lead to cell proliferation, survival, angiogenesis, and metastasis via four possible pathways; the phosphatidylinositol 3-kinase (PI3K)/Akt/mammalian target of rapamycin (mTOR) pathway, Ras/Raf/mitogen-activated protein kinase (MAPK) pathway, nitric oxide synthase (NOS), and prostaglandin (PG) involving phospholipase C*γ* (PLC*γ*)/protein kinase C (PKC) pathway and signal transducer and activator of transcription (STAT) pathway.

The growth factor receptors respond to their specific ligands and mediate tumorigenic activity through variety of signaling pathways (Figure 1). In case of NSCLC it was shown that epidermal growth factor receptor (EGFR) is overexpressed and specific somatic mutations occurred in their intracellular domain which may influence prognosis and significantly related to stage, survival, and chemotherapy response [11]. There are four main intracellular signaling pathways involved in the activation of EGFR: Ras/mitogen-activated protein kinase (MAPK), phosphatidylinositol 3-kinase (PI3K)/Akt, phospholipase C*γ* (PLC*γ*), protein kinase C (PKC), and signal transducer and activator of transcription (STAT) [12]. Like EGFR, vascular endothelial growth factor (VEGF) also plays a critical role in tumorigenesis, and these two pathways are linked together [13]. VEGF is associated with neovascularization and influences progression of NSCLC [14], and inhibition of VEGF or its receptor (VEGFR) correlate with impaired cell-cycle distribution, overall survival, and chemotherapy response [15, 16]. VEGFR mediates its angiogenic effect through PI3K/PLC*γ* pathway [17]. Enhanced activity of platelet derived growth factor (PDGF) is associated with tumorigenesis and angiogenesis [18] and inhibition of PDGF receptor (PDGFR) impede tumor growth by impairing periendothelial cell recruitment in A549 NSCLC cell line [19]. Coexpression of PDGF-B and VEGFR-3 is associated with poor survival in NSCLC patients [20]. The fibroblast growth factor (FGF) family comprises 22 ligands [21] with numerous isoforms of FGF receptor tyrosine kinases generated by messenger RNA splicing from 4 genes (FGFR1, FGFR2, FGFR3, and FGFR4) [22]. As reviewed by Semrad and Mack [23], FGFs and FGFRs play multiple potential mechanisms for tumor proliferation, survival, neoangiogenesis, and metastases in NSCLC. Signaling through FGFRs is mediated by PI3K/PLC*γ* and Ras/mitogen-activated protein kinase (MAPK) signaling cascades [23]. The insulin-like growth factor (IGF) pathway is linked to the deregulation of cell growth and metastasis, and its growth-promoting activities are mediated by the IGF-1R [22]. IGF-1R expression is associated with reduced disease-free survival (DFS) in NSCLC [24]. IGF-1R is a glycoprotein composed of two extracellular alpha subunits that preferentially bind IGF-1 and IGF-2 and insulin with a lesser affinity. The two intracellular beta subunits contain the tyrosine kinase domain responsible for activating the IRS/PI3K/AKT/mTOR pathway and the Ras/Raf/MAPK pathways that promote cell growth, transformation, migration, and survival [25, 26].

## 3. GFR Targeted Therapy for NSCLC

Downstream signaling of GFRs can be abrogated by (1) inhibition of receptor expression using gene therapy (antisense approach), (2) antagonistic monoclonal antibodies (mAb) that prevent binding of ligands to receptors, or (3) pharmacologic (low-molecular weight) receptor-selective tyrosine kinase inhibitors (RTKIs). MAb and TKI actions are the preferred approaches currently under intense clinical development [27].

### 3.1. Mechanism of Action of mAbs

**Figure 2:**
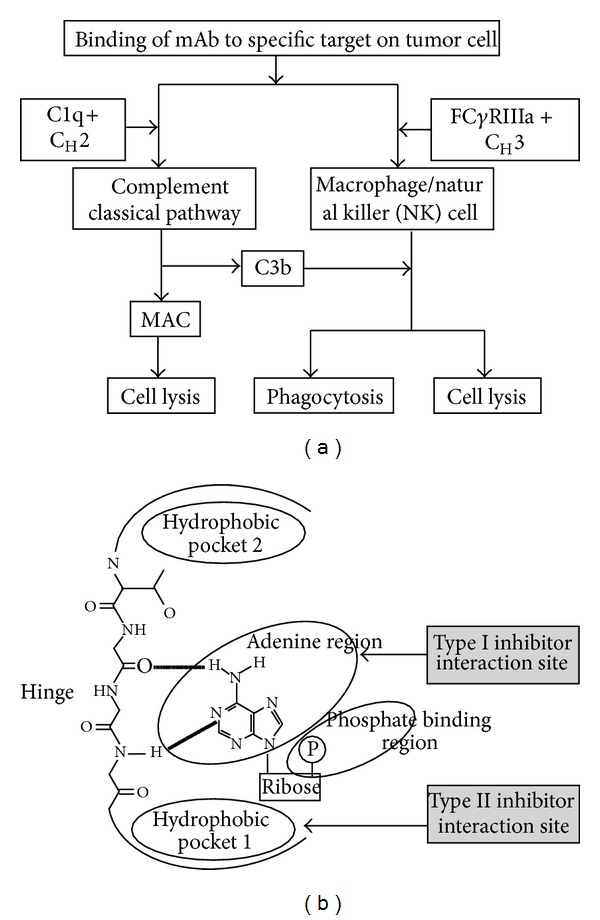
Growth factor receptor inhibition. (a) Immuno-modulatory mechanism for monoclonal antibodies. Binding of monoclonal antibodies (mAbs) to a specific target on a tumour cell can cause either complement-dependent cytotoxicity (CDC) by the interaction of C1q complement factor with the C_H_2 constant region of the mAb, which leads to the activation of complement classical pathway and induces the formation of a membrane-attack complex (MAC) for the lysis of tumour cells or antibody-dependent cellular cytotoxicity (ADCC) by the interaction of C_H_3 region of the mAbs with Fc*γ*RIIIa expressed by effector cells (macrophages or NK cells) which leads to phagocytosis by macrophages or undergo cytolysis by NK cells. C3b, which is generated during CDC, can facilitate phagocytosis and cytolysis through its interaction with macrophage or natural killer (NK) cell. This effect is termed as complement-dependent cell-mediated cytotoxicity (CDCC). (b) Small-molecule receptor tyrosine kinase inhibitor interaction to its specific site. Small-molecule tyrosine kinase inhibitors (TKIs) function as ATP analogues to compete with ATP for their binding site and block the receptor mediated downstream signaling.

In the early 1980s mAb was first applied for cancer treatment, about 10 years earlier than the development of small-molecule TKIs. Although early clinical trials with murine mAbs failed owing to their short half-life and limited activity, genetic engineering has made it possible to design chimeric mouse-human mAbs and humanized mAbs [28]. Among a number of these antibodies (Table 1), bevacizumab, a humanized mAb to vascular endothelial growth factor (VEGF), is currently approved for use in combination with chemotherapy in multiple countries for the treatment of patients with metastatic NSCLC [29]. The mAbs can confer their antitumor activity by two putative mechanisms. For the direct action, it can block the function of target signaling molecules resulting in inhibition of cell-cycle progression, regression of angiogenesis, induction of apoptosis, and internalization of receptors or it can be conjugated with toxins, cytokines, and even with small-molecule agents [28, 30]. The second or indirect mechanism of mAb is mediated by the immune system through complement-dependent cytotoxicity (CDC), complement-dependent cell-mediated cytotoxicity (CDCC), or antibody-dependent cellular cytotoxicity (ADCC) [31] (Figure 2(a)).

### 3.2. Mechanism of Action of RTKIs

Most small-molecule kinase inhibitors, discovered to date, are ATP mimetic that compete with ATP for the ATP-binding cleft of the kinase domain of the receptor. The binding may differ for the inhibitors, and at least three types of binding have been found. Among them the first is recognition of the active conformation of a kinase by the inhibitor and mimic the hydrogen bond that is normally formed by ATP [32]. These inhibitors, for example, sunitinib, usually present one to three hydrogen bonds to the ATP-binding site [32, 33]. The second type of inhibitors modulates kinase activity and its downstream signaling in an allosteric way by using a hydrophobic pocket to (indirectly) compete with ATP [34]. Sorafenib belongs to this kind of kinase inhibitors group [35]. The third kind is covalent inhibitors that react with electron rich sulfur (S) atom of cysteine residue by sharing electrons at specific sites of the kinase which allows the inhibitor to block binding of ATP to the kinase and prevents its activation [36]. Example of this kind of inhibitor includes vandetanib [37] (Figure 2(b)).

It is quite impossible to predict which of these two approaches can be more effective; moreover there is no clear difference in the spectrum of cancers targeted by mAbs and small-molecule inhibitors. One advantage of TKIs over mAbs is that they are administered orally, making them ideal for chronic maintenance therapy although they have a shorter half-life than mAbs. Furthermore these antibodies induce downregulation of surface receptors in addition to blocking ligand binding to receptors as well as incite antibody-dependent cell-mediated cytotoxicity [38].

## 4. Inhibition of Multiple Signalling by RTKIs

### 4.1. VEGFR/PDGFR Dual Inhibition

#### 4.1.1. Sorafenib

Sorafenib (Bay 43-9006, Nexavar, Bayer; Leverkusen, Germany) can block the phosphorylation of VEGFR, PDGFR, Raf, and stem cell factor receptor (KIT). In phase II studies, single-agent sorafenib prolonged progression-free survival (PFS) [39]. Several studies about combination of sorafenib with chemotherapeutic agents reported no beneficial effects. Placebo-controlled phase III trials of carboplatin/paclitaxel [40] and gemcitabine/cisplatin [41, 42] alone or with sorafenib have demonstrated no additional benefit of response rate, PFS, or OS. Patients with squamous histology were found to be more vulnerable in mortality index. But the combination of sorafenib with another EGFR tyrosine kinase inhibitor erlotinib proved promising clinical activity in patients with advanced NSCLC with an acceptable safety profile. A multicenter phase II study of erlotinib and sorafenib in patients with adenocarcinoma (72%), squamous (10%), large cell (12%), and NSCLC NOS (6%) histology reported an OS of 10.9 months with its adverse events which included fatigue (16%), hand-foot skin reaction (16%), rash (16%), diarrhea (14%), and hypophosphatemia (42%) [43]. Another phase II study (patients with squamous histology were excluded) demonstrated the higher activity of sorafenib/erlotinib combination than sorafenib/gemcitabine combination with a higher 1-year survival rate for first group patients [44]. Benefit for the combination of erlotinib/sorafenib over single-agent erlotinib with respect to PFS (3.38 months for sorafenib/erlotinib versus 1.94 months for placebo/erlotinib) and OS (8 months for sorafenib/erlotinib versus 4.5 months for placebo/erlotinib) has also been reported [45]. According to Adjei et al. [46], in patients with advanced NSCLC who achieve a prolonged response or stable disease with sorafenib given as a single agent or as part of a combination regimen, sorafenib treatment could be continued until disease progression without major long-term safety or tolerability problems. These studies can conclude that combination of erlotinib and sorafenib would take NSCLC therapy into a plateau.

#### 4.1.2. Sunitinib

Sunitinib (SU11248, Sutent, Pfizer; New London, CT, USA) is an oral, small-molecule, and multitargeted receptor tyrosine kinase (RTK) inhibitor of VEGFR-2 (Flk-1), PDGFR-*β*, and KIT, rearranged during transfection (RET), colony-stimulating factor 1 receptor (CSF-1R), and FMS-like tyrosine kinase-3 receptor (FLT3) [47]. Phase II studies of sunitinib on a continuous daily dosing (CDD) schedule suggested sunitinib as a safe and potential therapeutic agent for patients with previously treated advanced NSCLC of all histologies, though single-agent sunitinib was associated with a few manageable hematologic and neurologic abnormalities [48, 49]. Combinations of sunitinib with other agents were studied in order to gain high efficacy. But the results were not so satisfactory because of toxicities. A phase III trial revealed sunitinib plus erlotinib did not improve OS compared with erlotinib alone (9.0 months versus 8.5 months) in patients with NSCLC of nonsquamous (about 82% adenocarcinoma) histology, but the combination was associated with a statistically significant longer PFS (3.6 months versus 2.0 months) and greater overall response rate (ORR) (10.6% versus 6.9%) [50]. That trial also revealed a greater grade 3 or higher toxicities with combined therapy. Earlier a study about sunitinib with bevacizumab plus paclitaxel/carboplatin was discontinued because of toxicities including neutropenia, thrombocytopenia, leukopenia, and fatigue on its first phase [51]. However Blumenschein Jr. et al. [52] suggested on their randomized, double-blind, and multicenter study that a dosage of sunitinib concurrently with erlotinib was tolerable in subjects with platinum-refractory NSCLC of adenocarcinoma (70.0%), squamous cell (26.7%), and large cell (3.3%) histology, but the adverse effects were not totally removed with diarrhea (76.9%), fatigue (61.5%), and decreased appetite (53.8%) being the most frequent adverse events.

#### 4.1.3. Cediranib

Cediranib (AZD2171, Recentin, AstraZeneca; Wilmington, DE, USA) is an oral TKI that targets VEGFR-1, VEGFR-2, VEGFR-3, PDGFRs, and c-kit [53]. Cediranib as a monotherapy was generally well tolerated and showed encouraging antitumor activity in a phase I, dose escalation, and pharmacokinetic study in patients with advanced solid tumor [54]. Phase I data suggested that cediranib could be combined with standard doses of cisplatin and gemcitabine in patients with advanced NSCLC with promising activity but predictable and manageable toxicity [55]. Based on these data, a randomized phase II/III trial of carboplatin/paclitaxel plus cediranib or placebo as first-line therapy for advanced NSCLC was initiated [56]. Although PFS favored the combination of chemotherapy plus cediranib regardless of patient's histology, the study encountered excessive toxicities including hypertension, hypothyroidism, hand-foot syndrome, hypoalbuminemia, and an imbalance in treatment related deaths (10 and 2 for chemotherapy plus cediranib and chemotherapy plus placebo, resp.). A randomized phase II study of gemcitabine and carboplatin with or without cediranib as first-line therapy in advanced NSCLC of squamous (15.5% versus 27.6%), adenocarcinoma (37.9% versus 55.2%), and other (46.6% versus 17.2%) histologies did not meet its primary endpoint of overall response rate (ORR) (19% versus 20%) but met its secondary endpoint of 6-month progression-free survival (48% versus protocol-specified threshold of at least 40%) with more grade 3 plus nonhematologic adverse events experienced by cediranib group [57].

#### 4.1.4. Linifanib

Linifanib (ABT-869, Abbott; Abbott Park, IL, USA) targets VEGFR-1, VEGFR-2, VEGFR-3, and PDGFR [58]. Phase I study showed that linifanib had a favorable safety profile with minimal dose limiting toxicities (DLTs) and median PFS of 5.7 months [59]. Another phase II trial of linifanib in 139 (122 nonsquamous and 17 squamous histology) patients with advanced nonsmall cell lung cancer showed that it was active in advanced nonsmall cell lung cancer as second- or third-line therapy. The study also reported a median PSF of 3.6 months, median OS of 9.0 months with the adverse events, comprising fatigue (42%), decreased appetite (38%), hypertension (37%), diarrhea (32%), nausea (27%), palmar-plantar erythrodysesthesia (24%), and proteinuria (22%) [60]. Comparatively low dose linifanib can be a potent small-molecule inhibitor against NSCLC though study about its combination with other targeting agents is limited.

#### 4.1.5. Axitinib

Axitinib (AG-013736, Pfizer; New London, CT, USA) is an oral TKI with activity against VEGFR, PDGFR, and c-kit [61]. In vitro, axitinib enhances efficacy of chemotherapeutic drugs (topotecan and mitoxantrone) via inhibiting the drug transport function of adenosine triphosphate- (ATP-) binding cassette subfamily G member 2 (ABCG2) and reversed ABCG2-mediated multidrug resistance (MDR) [62]. In a phase I trial study Kozloff et al. [63] demonstrated that administration of axitinib with paclitaxel/carboplatin or gemcitabine/cisplatin regimens was well tolerated with improved clinical efficacy. They have also reported no grade ≥3 haemoptysis among 12 patients with squamous cell NSCLC out of total 49 patients suggesting clinically working further with this drug.

#### 4.1.6. Motesanib

Motesanib (AMG 706; Amgen, Thousand Oaks, CA, USA) is an orally administered potent small molecule, which targets VEGFR-1, VEGFR-2, VEGFR-3, PDGFR, and KIT [64]. In human NSCLC xenograft models, motesanib has been proved as a potent antitumor agent and that it had enhanced activity when combined with cisplatin or docetaxel [65]. But clinical trials of combination have different results. Several phase III trials have failed to achieve their primary endpoint of OS. A randomized, double-blind phase III study of motesanib plus carboplatin/paclitaxel in 1090 patients (of those, 890 had adenocarcinoma) with advanced NSCLC demonstrated the combination did not significantly improve OS over carboplatin/paclitaxel alone, with a median OS of 13 months and 11 months and median PFS of 5.6 months and 5.4 months for carboplatin/paclitaxel plus motesanib and carboplatin/paclitaxel alone, respectively [50]. The grade ≥3 and grade 5 adverse effects were higher with motesanib treatment. Similarly another MONET1 (Motesanib NSCLC Efficacy and Tolerability) study reported improvement on PFS but no OS benefit with increased toxicity [66].

### 4.2. VEGFR/EGFR Dual Inhibition

#### 4.2.1. Vandetanib

Vandetanib (AZD6474, Zactima, AstraZeneca; Wilmington, DE, USA) is a multitargeted tyrosine kinase inhibitor (TKI) that inhibits VEGFR, EGFR, and RET [67]. Single-agent vandetanib has antitumor activity with an acceptable safety and tolerability profile [68]. Combinations of vandetanib with chemotherapeutic agents are being evaluating for better efficacy as expected combination of vandetanib with paclitaxel and/or carboplatin have increased PFS [69]. But the adverse effects of combining therapy are still in concern. Three phase III studies also provide contradictory concepts. The ZEAL trial (*N* = 534), which compared vandetanib plus pemetrexed versus placebo plus pemetrexed, failed to meet its primary endpoint of prolonged PFS [70]. Whereas the ZODIAC trial, which compared vandetanib plus docetaxel (*N* = 694; of those 412 had adenocarcinoma, 184 had squamous, and 98 had other histology) versus placebo plus docetaxel (697 patients; of those 417 had adenocarcinoma, 160 had squamous, and 120 had other histologies) [71], the ZEPHYR trial (924 patients; of those 726 had adenocarcinoma, 102 had squamous, and 96 had other histologies) evaluated vandetanib in patients with advanced NSCLC, and prior EGFR TKI therapy demonstrated significantly improved PFS for the combination but did not significantly improve OS [72]. In addition both studies showed higher incidence of adverse effects for vandetanib group.

#### 4.2.2. XL647

XL647 (EXEL 7647) is an oral TKI with activity against EGFR, HER2, VEGFR, and EphB4 [73]. XL647 inhibits EGFR phosphorylation as well as phosphorylation of downstream signals (AKT, ERK). XL647 demonstrated antitumor activity in patients with EGFR-activating mutations and adenocarcinoma histology (41 patients) in a phase II study with most common adverse effects being diarrhea, nausea, and fatigue [74]. XL647 has also showed to be active against cells containing the T790M mutation, which have been associated with emerging resistance to first-generation EGFR TKIs such as gefitinib or erlotinib [75]. But in a phase II study of 41 patients among those 38 patients was with adenocarcinoma, 1 with squamous and 2 with undefined histology; it has failed to meet the primary endpoint of response rate in patients with T790M with significantly worse progression-free survival [76]. Further studies are required to develop this agent as a potential second-generation TKI to delay or overcome acquired resistance to first-generation EGFR TKIs.

### 4.3. VEGFR/FGFR Dual Inhibition

#### 4.3.1. Brivanib

 Brivanib (BMS-540215, Bristol-Myers Squibb; New York, NY, USA) is a small-molecule TKI, formulated as an orally administered l-alanine ester prodrug, brivanib alaninate [77]. It is the first-selective dual inhibitor of FGF and VEGF signaling [78], and preclinical studies have demonstrated that dual inhibition of FGF and VEGF signaling by brivanib has strong antiangiogenic effects [79]. In phase I study in patients with advanced or metastatic solid tumors, brivanib demonstrated promising antiangiogenic and antitumor activity and manageable toxicity with most frequent serious toxic effects recorded being nausea, pyrexia, aspartate aminotransferase (AST) or alanine aminotransferase (ALT) elevations, and thrombocytopenia [80, 81]. Synergistic activity of ixabepilone plus brivanib has suggested a promising anticancer activity both in vitro and in vivo [82]. 

### 4.4. VEGFR/PDGFR/FGFR Triple-Target Inhibition

#### 4.4.1. Nintedanib

Nintedanib or BIBF 1120 (Boehringer Ingelheim; Ingelheim, Germany) is an oral small-molecule triple kinase inhibitor which inhibits VEGFR, PDGFR, and FGFRs and members of the Src family and flt-3 [83]. Single-agent treatment of nintedanib (BIBF 1120) in advanced NCSLC was well tolerated, with median PFS of 6.9 weeks and median overall survival (OS) of 21.9 weeks [84]. 73 patients (40 with adenocarcinoma, 9 with large cell carcinoma, 17 with squamous cell carcinoma, and 7 undefined histology) were enrolled, and, according to this phase II double-blind study, drug-related adverse effects were nausea (57.5%), diarrhoea (47.9%), vomiting (42.5%), anorexia (28.8%), abdominal pain (13.7%), and reversible elevation of alanine transaminase (13.7%) and aspartate aminotransferase (9.6%). This drug also has proved its safety and well tolerability on its phase I trials in combination with chemotherapy. Phase I trial of 26 patients (7 with adenocarcinoma, 3 with squamous, 1 with large cell carcinoma, 1 with adenosquamous, and 14 with unspecified histology) with recurrent advanced-stage NSCLC receiving BIBF 1120 in combination with standard-dose pemetrexed was tolerable, with promising signs of efficacy [85]. For combination with paclitaxel and carboplatin, nintedanib demonstrated an acceptable safety profile in a phase I trial [86]. These findings indicate nintedanib as a potential triple kinase inhibitor and as a candidate for further study.

#### 4.4.2. Pazopanib

Pazopanib (GW786034, GlaxoSmithKline; London, UK) is another triple targeted TKI targeting VEGF, PDGFR, and FGFR [87]. Phase II study of pazopanib monotherapy in 35 patients (23 with adenocarcinoma, 4 with squamous, and 8 with other histology) with stage I/II NSCLC demonstrated well tolerability with 86% patients who achieved tumor volume reduction after pazopanib treatment [88]. Clinical data with pazopanib activity in advanced disease stage and in combination with other drugs are strikingly limited. A phase I study of pazopanib plus gemcitabine combination therapy in patients with advanced solid tumors explored that the combination was tolerable, and there was no apparent pharmacokinetic interaction [89].

## 5. Increasing Potential of TKIs

A rationale for combining molecular targeted therapies might confer additional clinical benefit and eventually reduce toxicities compared with standard chemotherapy in patients with advanced disease. Certainly cancer treatment strategies have been blessed with targeted therapy, but, for multitargeted TKIs, failure to respond is more frequent. Searching the reasons behind such failure of multitargeted therapies is a great scientific issue nowaday. Regarding toxicity, it is still a question whether the therapeutic indices of these treatment strategies are sufficiently enhancing. Clinical trials for vandetanib, sorafenib, sunitinib, and cediranib to treat NSCLC were all stopped early due to futility or toxicity issues [40, 42, 51, 56]. Resistance occurring from mutation or utilization of cross-talk and redundancy in signaling pathways by the tumor cells remains the rule rather than the exception. But it is not clear whether the failure of multitargeted RTKIs to treat NSCLC arise from resistance as found for the first generation TKIs like gefitinib, erlotinib or crizotinib [90, 91]. Identifying most responsive patients to a specific agent is crucial for the clinical development of targeted agents in patients with NSCLC. Biomarkers, such as EGFR, KRAS, and BRCA1, have been identified to be associated with patient prognosis and/or response to therapy [92]. In fact BATTLE (Biomarker-integrated Approaches of Targeted Therapy for Lung Cancer Elimination) trial for erlotinib, vandetanib, erlotinib plus bexarotene, or sorafenib, based on relevant molecular biomarkers, establishes a new paradigm for individualized treatment approach in lung cancer clinical trials [93]. But it is hard to find studies based on these biomarkers. Studies on population with specific GFR gene mutation are strikingly limited for multitargeted inhibitors. So far, only one phase II study of XL647 has been done on patients with a known sensitizing mutation of EGFR and confirmed antitumor activity of this small-molecule GFR inhibitor [74]. Patient selection irrespective to histology is suspected to be a reason for the failure of multitargeted NSCLC therapy. Histology has been emerged as a potential predictive factor for the outcome of patients with advanced NSCLC treated with EGFR inhibitors [94]. It has been found that, though vast majority of patients have failed to respond to lung cancer therapy, a minority of population, especially those with adenocarcinoma histology and never smokers, showed dramatic tumor reduction with symptomatic improvement [95, 96]. One of the possible reasons why targeted therapy of lung adenocarcinoma compares favorably with other histologies may be its distinct biological behavior [97]. For example, the expression of HER family members (HER-2), not EGFR expression, is correlated with the efficacy of gefitinib, and it has been found that adenocarcinomas of the lung are more likely to have increased immunohistochemical staining for HER-2 than squamous cell or other NSCLC histologies [96]. Histology should be a key factor for treatment choice in the routine care of patients with NSCLC.

## 6. Conclusion

Nevertheless, to date, a number of platinum-based regimens using as standard therapies have shown modest improvements in both overall survival and quality of life; they are associated with significant toxicities. Moreover our growing knowledge of molecular signaling that makes tumor cell defiant and the poor survival of patients with relapsed or progressive NSCLC reminds us to develop new drugs with better safety profile. Targeting either the VEGFR or EGFR signaling pathways has been clinically validated, and a number of drugs (e.g., bevacizumab, erlotinib, cetuximab, and gefitinib) are currently approved for treating advanced NSCLC. But the ability of solid tumors to develop multiple salvage and resistance pathways that allow them to circumvent inhibition of a single signaling pathway is becoming increasingly evident [98]. Resistance is less likely to arise if multiple regulatory pathways are being targeted at the same time. So, the development of multitargeted TKIs is in concern. Though there is an increasing research interest about these kinds of drugs, their therapy related adverse effects and safety remain in controversy. As we discussed, some clinical trial has been stopped early because of its toxicity issues, and some others could not meet their primary improvement of overall survival; there is still the need to explore more convenient, newer pathways as well as to put insight into coinhibition of existing pathways. It is important to pay more attention to the signaling pathways that are modified by the use of kinase inhibitors. Genomic landscapes for patient-specific kinomes should be provided to appropriately select patients who are most likely to be benefited from RTK-inhibition therapy. The future for growth factor receptor targeted therapy for NSCLC certainly looks bright.

## Figures and Tables

**Table 1:** GFR targeted monoclonal antibodies (mAbs) for NSCLC therapy.

mAb name	Targeted GFR	Source	Current developed phase	Ongoing trial^a^
Cetuximab	EGFR	Chimeric	III	NCT00408499 NCT00397384 NCT00946712 NCT00533949 NCT00368992 NCT01059188 NCT00867009 NCT00842712

Cixutumumab	IGF-1R	Human	I/II	NCT00955305 NCT01232452 NCT01263782 NCT00887159

EMD 72000 (Matuzumab)	EGFR	Humanized from mouse	II	—

Figitumumab (CP-751,871)	IGF-1R	Human	II	NCT00728390

Ganitumab (AMG 479)	IGF-1R	Human	I	NCT01327612

Necitumumab (IMC-11F8)	EGRF	Human	III	NCT00982111 NCT00981058 NCT01769391

Nimotuzumab	EGFR	Humanized from mouse	II	NCT01498562 NCT01393080

Olaratumab (IMC-3G3/LY3012207)	PDGFR-*α*	Human	II	NCT00918203

Panitumumab	EGFR	Human	I	NCT00979212 NCT01042288 NCT01038037

Ramucirumab (IMC-1121B)	VEGF-2	Human	III	NCT01160744 NCT01168973 NCT01703091

Trastuzumab	HER-2	Humanized from mouse	II	NCT00847366 NCT01148849

^a^ http://www.clinicaltrial.gov/, access date March 16, 2013.
